# Supplementary material for: Local Bayesian Dirichlet mixing of imperfect models
Source: Sci Rep. 2023 Nov 10;13:19600. doi: 10.1038/s41598-023-46568-0 (PMC10638441; doi:10.1038/s41598-023-46568-0)
Supplement: Supplementary file 1 — Supplementary Figure S1. [file 41598_2023_46568_MOESM1_ESM.pdf]

Supplementary Information for "Local Bayesian Dirichlet mixing of imperfect models"

V. Kejzlar[1], L. Neufcourt[2], W. Nazarewicz[3]

[1]Mathematics and Statistics Department, Skidmore College, Saratoga Springs, New York 12866, USA [2]FRIB Laboratory, Michigan State University, East Lansing, Michigan 48824, USA [3]Department of Physics and Astronomy and FRIB Laboratory, Michigan State University, East Lansing, Michigan 48824, USA

This supplementary information contains:

- Supplementary figures

I. SUPPLEMENTARY FIGURES

The results of LBMM+GLD in terms of the local weights at their posterior mean are shown in Fig. S1.

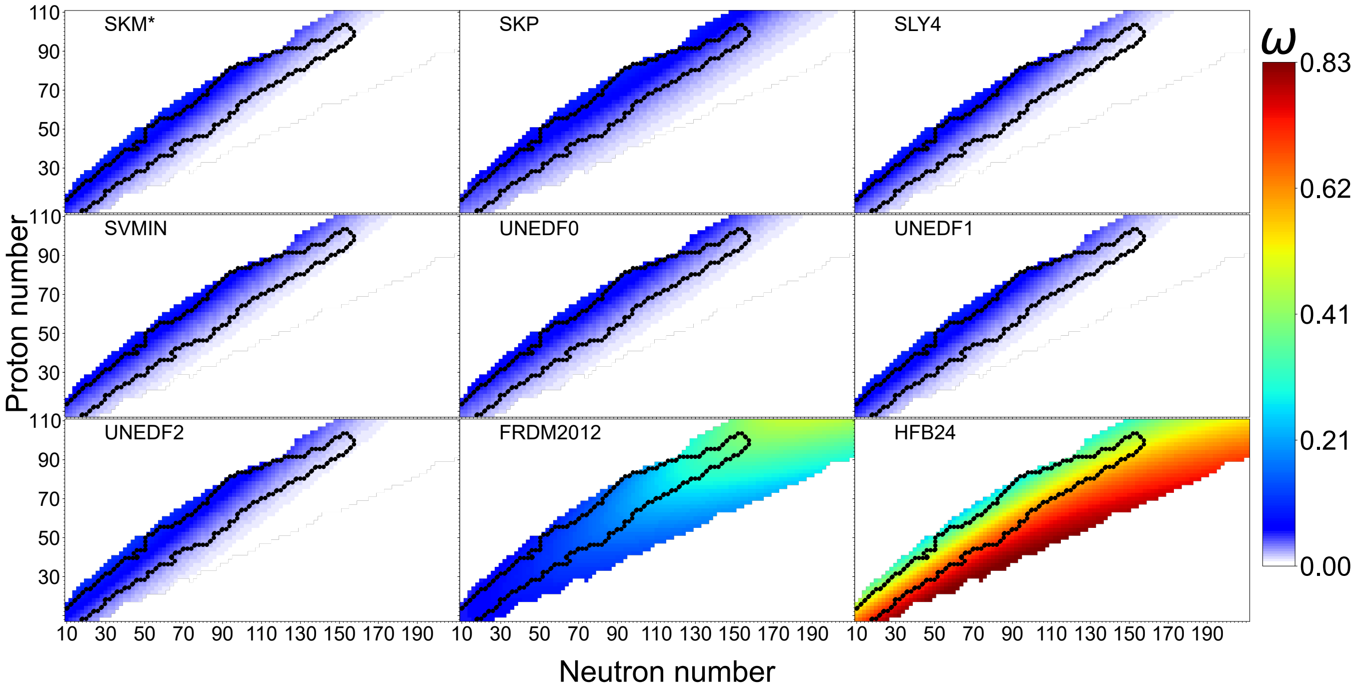

FIG. S1. Posterior means of the local model weights in the LBMM+GLD variant across the nuclear landscape.
